# An Unusual Presentation of Granulomatosis With Polyangiitis (Wegener's) After SARS-CoV-2 Infection

**DOI:** 10.7759/cureus.50088

**Published:** 2023-12-06

**Authors:** Daniele Romanello, Marta Giacomelli, Ilaria Coccia, Paolo Lido, Sara Rotunno

**Affiliations:** 1 Internal Medicine, Ospedale San Pietro Fatebenefratelli, Rome, ITA; 2 Internal Medicine, University of Rome "Campus Bio-Medico", Rome, ITA; 3 Internal Medicine, University of Rome Tor Vergata, Rome, ITA; 4 Clinical Sciences and Translational Medicine, University of Rome Tor Vergata, Rome, ITA

**Keywords:** differential diagnosi, anca associated vasculitis, pulmonary cavitary lesion, covid-19 related issues, unusual case, wegener’s granulomatosis, granulomatosis with polyangiitis (gpa)

## Abstract

In this article, we present an unusual case of granulomatosis with polyangiitis (GPA) in a 41-year-old man. The initial presentation of the disease was atypical, with persistent fever, cough, and fatigue, accompanied by elevated inflammatory markers in association with a large, solitary lung lesion observed at the chest X-ray. Despite the presence of an initial radiological picture suggesting pneumonia, the lack of response to antibiotics necessitated a more in-depth evaluation. The diagnosis was confirmed through a lung biopsy and serological tests positive for anti-neutrophil cytoplasmic antibodies (c-ANCA). GPA is an anti-neutrophil cytoplasmic antibody (ANCA)-associated vasculitis, a systemic autoimmune disease characterized by necrotizing granulomatous inflammation and pauci-immune small vessel vasculitis. This case posed diagnostic challenges due to the atypical presentation, initially mistaken for a respiratory tract infection versus cancer. However, the lack of improvement with antibiotics and persistent inflammation raised suspicions of an underlying complex condition. The diagnosis was confirmed through a lung biopsy and positive c-ANCA serological tests. The patient had reported a prior SARS-CoV-2 infection, raising questions about the possible connection between COVID-19 and GPA, as suggested by previous studies. The diagnostic workup ruled out common and rare pulmonary infections, autoimmune diseases, and neoplasms. However, the presence of positive c-ANCA antibodies was pivotal for the GPA diagnosis. Treatment involved the use of high-dose corticosteroids and rituximab to suppress the autoimmune response. Early diagnosis and timely treatment are essential for improving outcomes in patients with GPA.

## Introduction

This article was previously presented as an abstract at the 28th National FADOI Meeting in May 2023. Granulomatosis with polyangiitis (GPA), previously called Wegener’s granulomatosis, is an idiopathic, systemic inflammatory disease characterized by necrotizing granulomatous inflammation and pauci-immune small vessel vasculitis of the upper and lower respiratory tract and kidneys. According to the Modern Nomenclature of Systemic Vasculitis (Chapel Hill Consensus Conference in 2012), it is classified as an anti-neutrophil-cytoplasmic antibody (ANCA)-associated vasculitis (AAV). This classification also includes microscopic polyangiitis (MPA) and eosinophilic granulomatosis with polyangiitis (EGPA, or Churg Strauss syndrome) [1]. The disease typically affects the upper and lower respiratory tracts, with initial manifestations often being local. However, the clinical course can become severe, and approximately 80% of patients develop renal complications, presenting as glomerulonephritis. Aural involvement, detected in 25% to 40% of patients, includes conditions such as otitis media with effusion, chronic suppurative otitis media, sensorineural hearing loss, and vertigo attributed to cochlear vasculitis. Patients with generalized GPA may also experience ocular manifestations, such as ischemic optic neuropathy, optic nerve edema, optic atrophy, corneal melt, and unilateral or bilateral vision loss (20% to 50%) [2,3]. Additional involvements include the musculoskeletal system, presenting with myalgias, arthralgias, and arthritis [4-6]. Cutaneous manifestations may appear as palpable purpura, ulcers, vesicles, papules, and subcutaneous nodules [7,8]. Cardiac involvement may manifest as pericarditis and valvular lesions [9], while neurological symptoms can include mononeuritis multiplex, neuropathy, stroke, seizures, cerebritis, or meningitis [10]. Generalized symptoms like fever and malaise may also be present [11]. In this paper, we present an unusual case of granulomatosis with polyangiitis in a 41-year-old man.

## Case presentation

**Figure 1:**
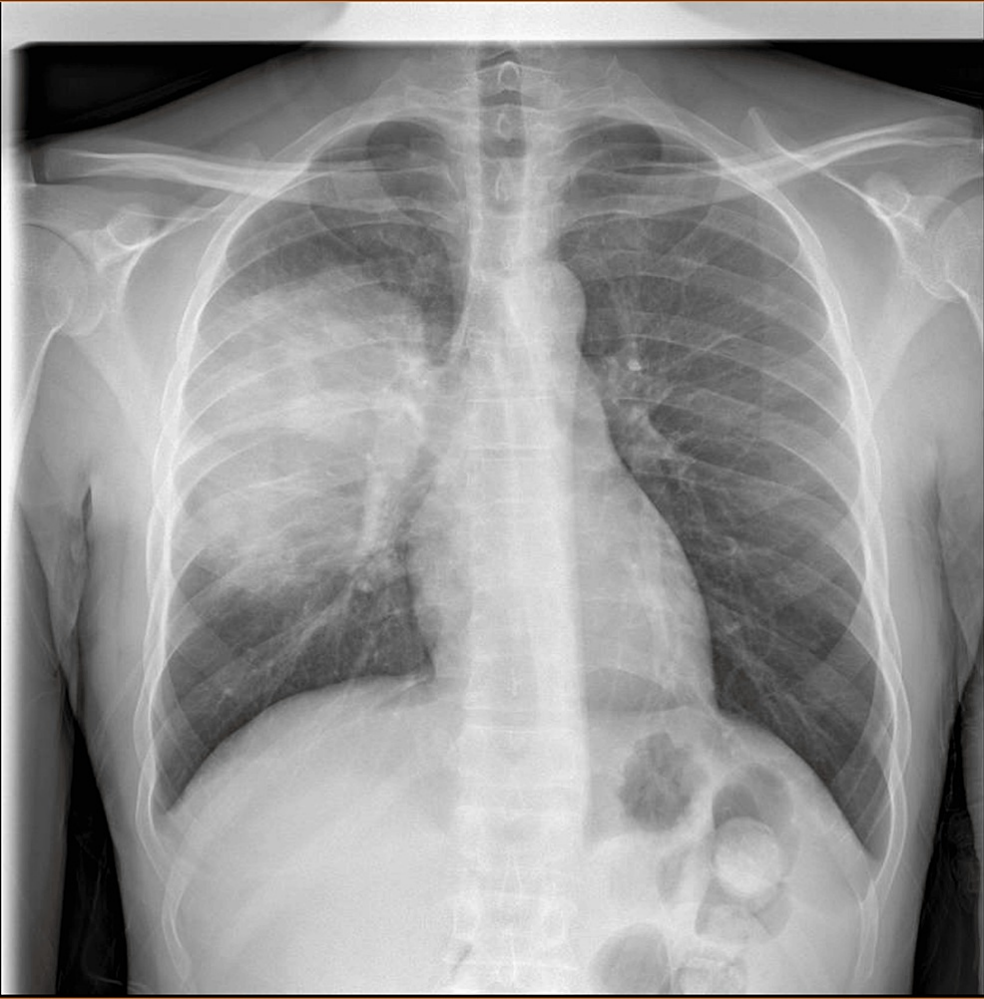
Chest X-ray front A coarse area of parenchymal hypodensity with rounded margins measuring 13 x 11 cm, without an air bronchogram.

**Figure 2:**
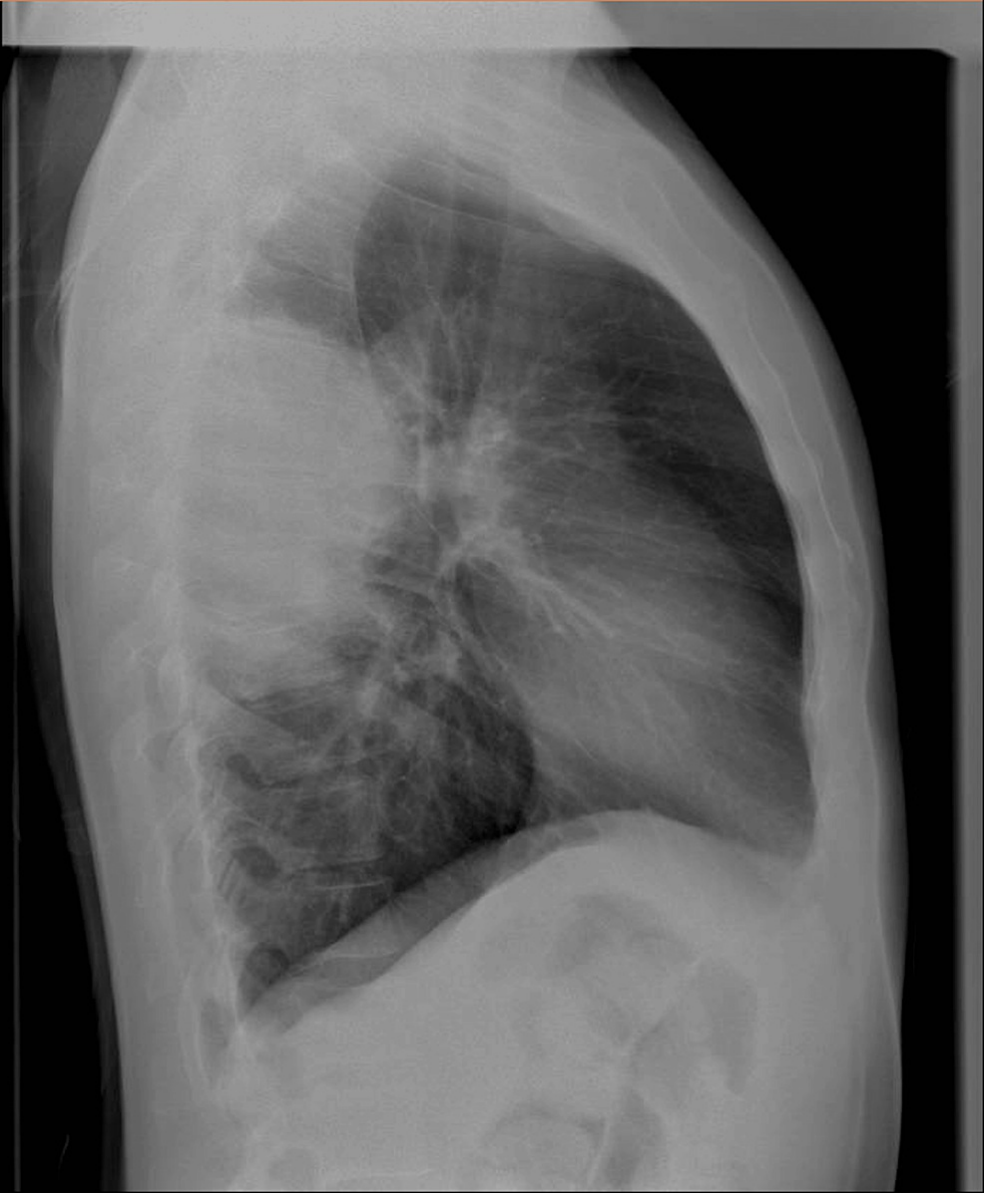
Chest X-Ray lateral Same lesion, lateral view. The lesion demonstrates a greater extension on the posterior aspect, which comes into contact with the pleura.

A previously healthy 41-year-old Caucasian male was admitted to the hospital due to a persistent and antibiotic-resistant fever, cough, and sore throat. At home, he was treated with levofloxacin (500 mg orally once daily) for seven days and ibuprofen without improvement. The initial laboratory investigation revealed mild anemia (hemoglobin 10.6 g/dL), neutrophilic leucocytosis, reactive thrombocytosis, elevated transaminase levels, and increased C-reactive protein (CRP) levels (160 mg/L). At admission, the nasopharyngeal swabs for SARS-CoV-2 were negative. Thorax examination showed symmetric lung expansion, increased vocal fremitus, and dullness on percussion at the lower part of the right chest and right basal crepitations. On examination, the blood pressure was 130/70 mm Hg, the pulse rate was 100 beats/min, and the SpO2 was 98% at room air. The patient denied any relevant past medical history, any exposure to tuberculosis, a history of drug abuse, tobacco or alcohol intake, or any occupational exposure to pollutants or allergens. The only medical history of note was a recent diagnosis (three months before) of SARS-CoV-2 infection that lasted one week, with three days of fever and a sore throat, and did not require hospitalization. Initial chest X-rays (Figure 1) revealed large parenchymal opacity (size of 13x11 cm) without an air bronchogram in the right upper/middle zone, suspicious of lobar pneumonia (Figure 2).

Initially, the patient was treated with piperacillin/tazobactam (4/0.5 g, three times daily IV) and clarithromycin (500 mg, twice daily IV), but the fever persisted. Pneumococcal and Legionella urinary antigens and molecular respiratory pathogens tests were negative. Urine analysis was normal, and urine culture and blood culture were performed. Sputum culture was positive for Candida albicans, sensitive to fluconazole. Therefore, due to the worsening clinical conditions of the patient despite the antibiotic therapy, intravenous fluconazole was added to the treatment.

**Figure 3:**
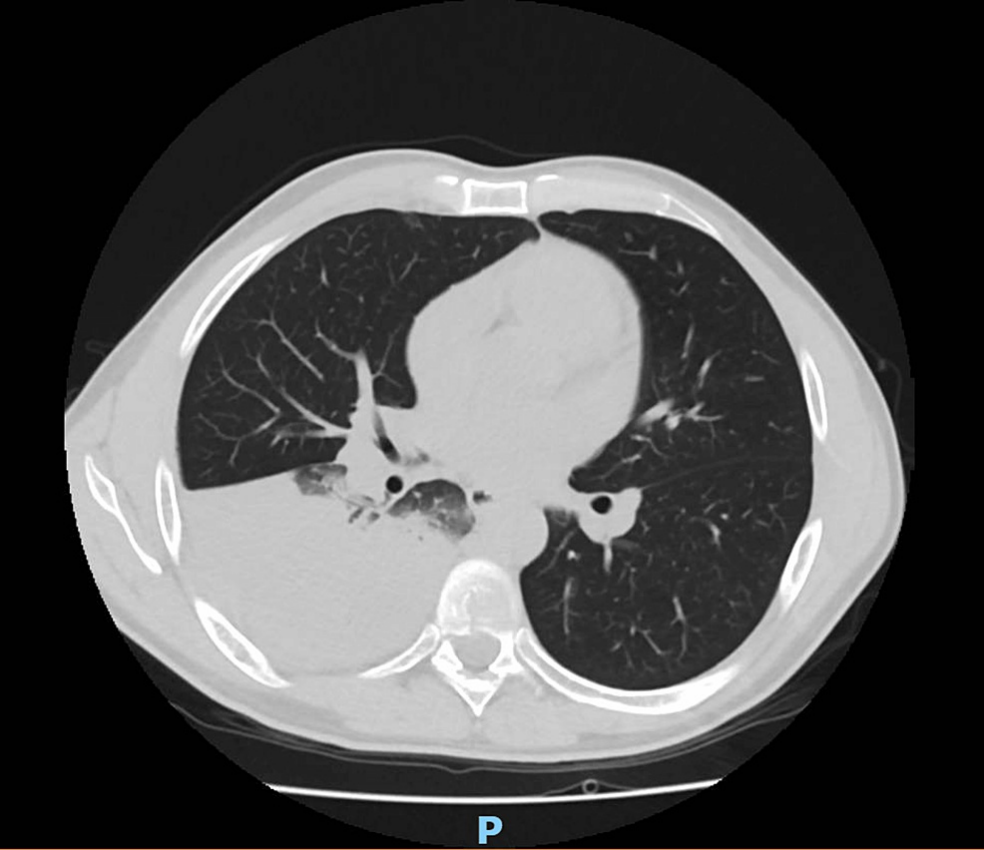
Axial CT scan Extensive parenchymal consolidation involving two-thirds of the right lower lobe without an air bronchogram.

**Figure 4:**
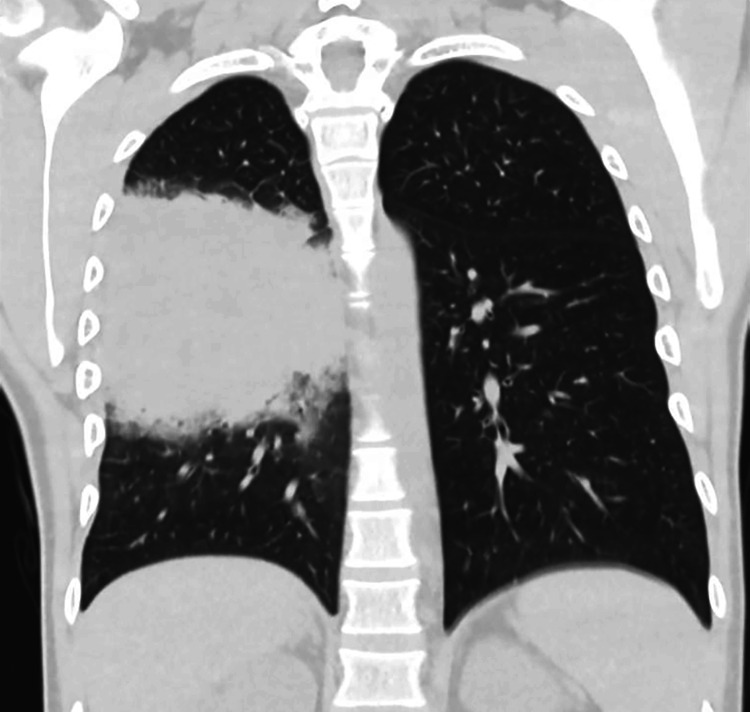
Coronal CT scan A lesion with heterogeneous density closely adheres to the subcostal pleura, causing compression and obliteration of the tributary bronchi.

CT of the chest with contrast showed parenchymal consolidation in the right lower lung lobe without a recognizable air-bronchogram strictly adhered to the right oblique fissure and to the subcostal pleura (Figure 3). The parenchymal consolidation had an irregular density with compression and obliteration of the posterior basal bronchi. The CT also showed a small, solid, non-calcified nodule in the right lower lung lobe (size 5 mm) and two additional nodules in the left lower lung lobe. The CT also revealed a small right-sided pleural effusion (Figure 4).

Investigations for infectious, malignant, and autoimmune diseases (cultures, serology for Herpes simplex virus, Epstein-Barr virus, cytomegalovirus, hepatitis B and C, Mantoux test, abdominal ultrasound, antineutrophil cytoplasm antibodies, and antinuclear factor) were performed. Oncomarkers (Alfa1 fetal protein, carcinoembrionic antigen (CEA), cancer antigen 19.9 (CA 19.9), cancer antigen 15.3 (CA 15.3), cancer antigen 125 (CA 125), and neuron-specific enolase [NSE]) were negative.

**Figure 5:**
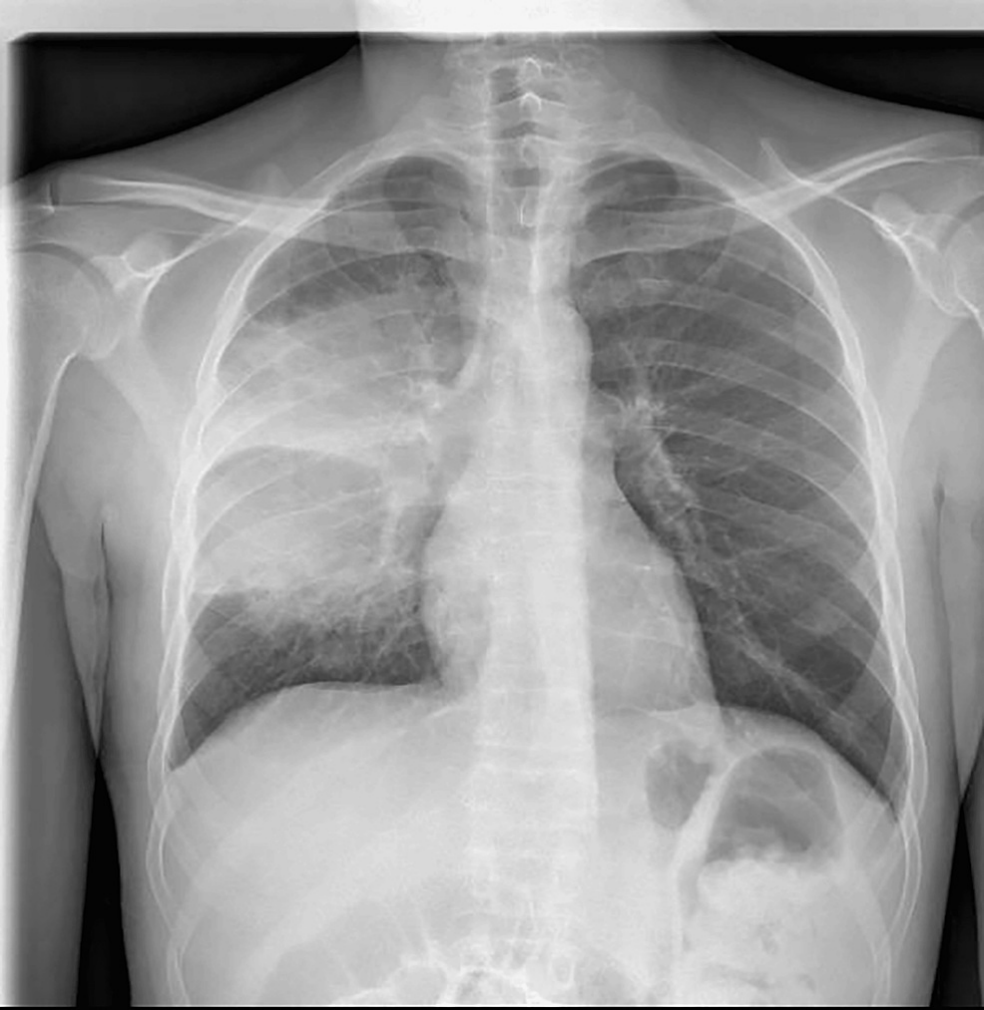
Chest X-ray after biopsy Chest X-ray for follow-up, performed after a CT-guided biopsy. The lesion shows an air-fluid level.

On day five of hospital admission, the patient experienced a new fever episode despite antibiotic treatment. Piperacillin/tazobactam and clarithromycin were replaced with Meropenem (1 g three times a day) and Linezolid (600 mg two times a day). The bronchoscopy did not reveal macroscopic alteration restricted to the capabilities of the procedure, with the absence of malignant cells in the cytological examination of BAL and no evidence of bacterial, viral, mycobacterial, or fungal infections in cultures. The total body CT was negative, with the exception of the known pulmonary lesions. A CT-guided percutaneous needle pulmonary biopsy was arranged to obtain a histological diagnosis. During the procedure, the patient developed a cough with hemoptysis. Control The chest X-ray showed the presence of an air bronchogram in the context of the known pulmonary opacity and a right-sided pleural effusion. After the lung biopsy, radiographs of the chest revealed a cavitary lesion with air-fluid levels (Figure 5).

In the microbiological examination of cavitary lesions, the galactomannan antigen on BAL yielded a negative result as well. On day 16 of hospital admission, the patient was discharged from the hospital. The results of histological examinations and tests for autoimmune diseases were pending. At the time of discharge from the hospital, the patient’s wife reported a previous history of epistaxis, which he has always attributed to capillary fragility. The autoimmune panel was positive for anti-proteinase 3 [PR3 cytoplasmic-antineutrophilic autoantibody (c-ANCA)] (588.70; negative < 20) and a negative antimyeloperoxidase antibody [MPO, perinuclear-antineutrophilic autoantibody (p-ANCA)] (2.9; negative < 20). The histological findings described increased neutrophilic granulocytes with signs of granulomatous vasculitis, suggesting granulomatosis with polyangiitis (Wegener’s).

The patient received high-dose corticosteroids through intravenous methylprednisolone at a daily dosage of 1000 mg for three consecutive days. Subsequently, the treatment was shifted to oral prednisone (1 mg/kg) tapered to 25 mg for oral administration in one month. After the high-dose therapy, the fever, asthenia, and cough resolved. Rheumatology was consulted for further evaluation and management. Following the definitive diagnosis of granulomatosis with polyangiitis, corticosteroids were tapered off, and the patient was started on Rituximab following a scheme of 1 infusion per week for four consecutive weeks. After the first infusion of rituximab, the prednisone dosage was reduced, but the patient developed scleritis of the right eye. After this, the cortisone levels were reduced to 25 mg, and the patient underwent an ophthalmologic follow-up. After 6 months, the patient underwent another cycle of infusion (1 week for 2 weeks consecutive). The prednisone was tapered to 2.5 mg per oral administration every 48 hours. At the last check-up, the patient did not present any symptoms. No follow-up radiological examinations were conducted at the current time.

## Discussion

In our case, the initial presentation was atypical. The symptoms were fever, cough, and fatigue, and the blood samples showed elevated inflammatory markers. The single chest X-ray lesion, the absence of cavitation, and the absence of kidney involvement didn’t lead to an immediate diagnosis of granulomatosis with polyangiitis.

The infectious hypothesis of lobar pneumoniae was the most probable. Pulmonary tubercolosis, Legionella, Pneumococcus, and Mycoplasma infections were excluded. Doubts arose when patients’ conditions didn’t improve despite first-line antibiotic therapy, first with Levofloxacin, then with Piperacillin/tazobactam and Clarithromycin, and there were no results even after an optimization of antibiotic therapy with a second line of antibiotics such as Meropenem and Linezolid. Indeed, he complained of greater fatigue and astenia, and inflammatory markers were steadily elevated.

Differential diagnosis from other diseases, such as neoplasia and immunological diseases, was necessary. Among the malignancies we considered were lymphomatoid granulomatosis, lymphomas, Castleman disease, and bronco-alveolar carcinoma. Among immune diseases, systemic lupus erythematosus, sarcoidosis, rheumatoid arthritis, and amyloidosis were investigated. On the one hand, a lack of response to antibiotics within 10 days, the presence of two pulmonary nodules controlateral to the main lesion, and the presence of numerous enlarged thoracic lymph nodes were in favor of malignancy diagnosis. On the other hand, the patient's young age, the presence of leukocytosis, high-grade fever, and the absence of weight loss were inconsistent with a malignancy diagnosis. Usually, malignancy affects elderly people, presenting weight loss and low-grade fever [12]. The patient's young age and the presence of nasal bleeding were indicative of an autoimmune disease.

A biopsy of the tissue involved showed necrotizing granulomas, and the c-ANCA positivity was crucial for diagnosis. C-ANCAs have a particularly strong correlation to granulomatosis with polyangiitis (up to 80% of patients, and possibly more of those with active disease, have these antibodies). Moreover, there were perplexing details, such as the initial absence of the typical pulmonary cavitary lesion of Wegener's granulomatosis, which only appeared in the chest X-ray after the lung biopsy, and the absence of kidney involvement with normal levels of creatinine, urea, and normal urinalysis.

The precise etiology of GPA remains elusive within the current medical understanding. The etiopathogenesis is attributed to anti-neutrophilic cytoplasmic antibodies (ANCA). The prevailing consensus implicates ANCA in driving the inflammatory processes observed in GPA. Aberrant immune-regulatory responses to environmental triggers, such as infection or autoantigens, result in an exaggerated production of Th1 and Th17 cytokines (interleukin 17, tumor necrosis factor, and interferon-gamma). This dysregulation subsequently fosters the formation of an inflammatory granulomatous vascular lesion. Infectious agents play a modulatory role in the clinical phenotype of the disease. The colonization by *Staphylococcus aureus* is postulated as a potential initiator of the inflammation witnessed in GPA. Additionally, associations with various viruses, including hepatitis C virus (HCV), cytomegalovirus (CMV), Epstein-Barr virus (EBV), and parvovirus, have been documented [13].

An important detail of the patient's history was that he had a recent diagnosis of a paucisymptomatic SARS-CoV-2 infection. Probably, SARS-CoV-2 infection initiated heightened levels of circulating cytokines and immune-cell hyperactivation, resulting in an imbalanced immune response, affecting not only the pathogen but also contributing to cellular, vascular injury, and multiorgan dysfunction. The cytokine-induced endothelial inflammation and vascular pathology associated with COVID-19 are extensively documented in post-mortem biopsies. Numerous cases have reported micro/macro thrombotic events in small, medium, and large vessels, as well as vasculitis affecting multiple organs [14-16].

Several studies have explored the spectrum of autoimmune-related manifestations in COVID-19 patients, ranging from organ-specific conditions (e.g., cutaneous vasculitis, immune thrombocytopenic purpura, transverse myelitis, Guillain-Barré syndrome) to systemic autoimmune and inflammatory disorders (e.g., systemic vasculitis, multisystem inflammatory syndrome [MIS], hemophagocytic lymphohistiocytosis [HLH], SLE) [17,18]. Additionally, autoantibodies such as antinuclear antibodies (ANA), anti-Ro/SSA, rheumatoid factor, lupus anticoagulant, and antibodies against interferon (IFN)-I have been reported in patients with COVID-19 [17,19]. Moreover, the identification of 28 human proteins with homologous regions to SARS-CoV-2 peptides suggests their potential role as autoantigens in COVID-19 patients with autoimmune conditions [17]. The precise pathophysiological correlation between SARS-CoV-2 infection and ANCA-associated vasculitis is not entirely clear and requires further investigation.

## Conclusions

In this document, we have presented an atypical onset of granulomatosis with polyangiitis in a 41-year-old man, confirmed by pulmonary biopsy and hematochemical determination. Early diagnosis of GPA requires a high index of suspicion in all patients, especially in atypical presentation cases. A careful differential diagnosis with infectious disease, neoplasia, and immunological disease led us to the correct diagnosis of granulomatosis with polyangiitis. Additionally, in the current setting of a global pandemic, suspicion for vasculitis should be high on the differential diagnosis in patients who are currently infected or have been infected with SARS-CoV-2. It is crucial to expedite the diagnostic process to ensure prompt and effective treatment and to prevent disease progression.
